# *Dnajb8*, a target gene of SOX30, is dispensable for male fertility in mice

**DOI:** 10.7717/peerj.10582

**Published:** 2020-12-21

**Authors:** Fengsong Wang, Shuai Kong, Xuechun Hu, Xin Li, Bo Xu, Qiuling Yue, Kaiqiang Fu, Lan Ye, Shun Bai

**Affiliations:** 1 School of Life Science, Anhui Medical University, Hefei, China; 2 Department of Urology, The First Affiliated Hospital of USTC, Division of Life Sciences and Medicine, University of Science and Technology of China, Hefei, China; 3 State Key Laboratory of Reproductive Medicine, Nanjing Medical University, Nanjing, China; 4 Reproductive and Genetic Hospital, The First Affiliated Hospital of USTC, Division of Life Sciences and Medicine, University of Science and Technology of China, Hefei, China; 5 Reproductive Medicine Center, The Affiliated Drum Tower Hospital of Nanjing University Medical School, Nanjing, China; 6 College of Veterinary Medicine, Qingdao Agricultural University, Qingdao, China

**Keywords:** Dnajb8, Male fertility, Spermatogenesis

## Abstract

**Background:**

The DNAJ family of molecular chaperones maintains protein homeostasis in mitotic and postmeiotic cells, especially germ cells. Recently, we found that the transcription factor SOX30 initiates transcription of *Dnajb8* during late meiosis and spermiogenesis in mouse testes.

**Methods:**

We used the CRISPR/Cas9 system to generate *Dnajb8* mutant mice and analyze the phenotype of the *Dnajb8* mutants.

**Results:**

Although*****Dnajb8* is an evolutionarily conserved gene, it is not essential for spermatogenesis and male fertility. We provide this phenotypic information, which could prevent duplicative work by other groups.

## Introduction

Mammalian spermatogenesis is a complicated cellular process during which diploid spermatogonial stem cells (SSCs) generate haploid spermatozoa. The process takes place in seminiferous epithelium of the testis and includes three successive developmental phases: spermatogonial proliferation and differentiation, two meiotic divisions to produce haploid round spermatids, and the differentiation of spermatids into sperm (Nishimura & L’Hernault, 2017). During spermatogenesis, the accurate regulation of protein folding and sorting is fundamental for the production of high-quality spermatozoa (Meccariello et al., 2014).

The DNAJ family is the largest molecular chaperone family and plays a key role in protein folding, trafficking, aggregation, homeostasis and conformation (Zarouchlioti et al., 2018). The human DNAJ proteins are divided into three subgroups based on a highly conserved domain structure containing a J domain that interacts with HSP70 (type III), a Gly/Phe-rich region (type II) and a cysteine-rich region (type I) (Cheetham & Caplan, 1998). Mutations in DNAJ proteins occur frequently in neurodegenerative diseases, such as cerebellar ataxia, distal hereditary motor neuropathy and Parkinson’s disease (Koutras & Braun, 2014). Nevertheless, DNAJ proteins has also been implicated in tumorogenesis and spermatogenesis (Kusumoto et al., 2018; Meccariello et al., 2014; Nishizawa et al., 2012). As there are functional differences among the DNAJ proteins, it is unclear whether deficiencies in each DNAJ protein could lead to male infertility.

DNAJB8, a member of the DNAJB family of proteins, plays a role in suppressing protein expression by interacting with HDACs (Hageman et al., 2010). A recent study also showed that DNAJB8 is a cancer-testis (CT) antigen carrying tumor-initiating ability (Nishizawa et al., 2012). Among mouse tissues, DNAJB8 is highly expressed in the testis, especially in postmeiotic germ cells (Nishizawa et al., 2012). Notably, we previously found that *Dnajb8* was directly regulated by the transcription factor SOX30, which controls the expression of a core set of postmeiotic genes (Bai et al., 2018). However, whether DNAJB8 affects spermatogenesis and male fertility is still not clear. Because in vitro spermiogenesis has still not been established, we generated a *Dnajb8* knockout (KO) mouse via Cas9/RNA-mediated gene targeting to understand the function of DNAJB8 in spermatogenesis.

## Materials and Methods

### Animals

Animals were housed in Laboratory Animal Center at University of Science and Technology of China under specific pathogen-free conditions with free access to food and water. All mice were treated humanely and euthanized by cervical dislocation to collected testis and epididymal samples for further analyses. The use of animals and the experimental design were approved by the Institutional Animal Care and Use Committees of the University of Science and Technology of China (No. 2019-N(A)-061), Hefei, China.

### Generation of an anti-DNAJB8 polyclonal antibody

DNAJB8 polyclonal antibody was prepared by ABclonal (Wuhan, China) and derived from rabbits. Recombinant fusion protein contained a sequence corresponding to a fragment (amino acids 116-132) of mouse DNAJB8. We have used this antibody for western blot.

### Generation of *Dnajb8* knockout mice with the CRISPR/Cas9 System

*Dnajb8 knockout mice were generated using CRISPR/Cas9 technology. The Dnajb8* exon containing the J-domain was targeted by sgRNAs. The sgRNA was synthesized by Genescript (Nanjing, China) and the sequences were as follows: 5′-ACCTGTCCCTGAGAACGGGG-3′and 5′-CATAGTAGTCGCAACCTAAC-3′. The Cas9 expressed vector pX330 was obtained from Addgene, linearized with NotI (New England Biolabs, USA), transcribed using T7 ULTRA Transcription Kit (Ambion AM1345, USA) and purified using MEGAclear™ Kit (Ambion AM1908, USA). Cas9 mRNA and sgRNAs were coinjected into fertilized eggs for KO mouse production. The pups were genotyped by genomic PCR followed by Sanger sequencing. After genotyping, the F0 mice went through serial mating to generate homozygous mutants.

### Genotyping

The genotype identification of offspring was completed by PCR amplification (primers: *Dnajb8* _F1: 5′-AGTCAAACAAACAGCCAAACTCAC-3′; R1: 5′-GTGACCGGAATAAATAACCTCCCA-3′; R2: 5′-TCGGAAACCTGCTTAAACTTCTTC-3′) and Sanger sequencing. The results of sequencing were analyzed by SnapGene.

### Fertility test

Every 8-week-old *Dnajb8*^+∕+^ and *Dnajb8*^−∕−^ male mouse was caged with two 8-week-old *Dnajb8*^+∕+^ female mice for at least 8 weeks. During the breeding test, the number of pups was counted at birth, and the average litter size for each mouse line was recorded.

### Histology analysis

Testes and epididymides from 10-week-old *Dnajb8*^+∕+^ and *Dnajb8*^−∕−^ male were fixed in Bouin’s solution overnight, dehydrated with increasing concentrations of ethanol (70%–100%) and embedded in paraffin. The tissue was cut into 5-µm-thick sections and mounted onto glass slides and followed by Hematoxylin and Eosin (H&E; Sigma-Aldrich, USA) staining. For sperm staining, epididymal sperm from the of 8-week-old male mice were extruded, fixed in 4% PFA at 4 °C overnight, and immobilized onto glass slides. The sections were stained with H&E. All sections were analyzed microscopically (LEICA DM2500, Germany).

### Sperm parameters analysis

Sperm parameters assays were performed using 10-week-old *Dnajb8*^+∕+^ and *Dnajb8*^−∕−^ male. Cauda epididymal sperm were extracted, incubated in PBS, fixed in 4% of PFA, followed by sperm counting using a hemocytometer. For sperm motility assays, cauda epididymal sperm were released into HTF medium at 37 °C and measured using computer-aided sperm analysis (CASA) system (Hamilton Thorne Biosciences, USA).

### Western blotting

Tissues and spermatozoa that separated from seminal plasma were rinsed with PBS and lysed in cold RIPA buffer supplemented with phosphatase inhibitor and protease inhibitor cocktail tablets. Lysates were incubated on ice and centrifuged at 13000 rpm for 15 min at 4 °C. The protein concentration was determined by the Bicinchoninic Acid (BCA) Assay (E11201, Vazyme, China) according to the manufacturer’s instructions. In total, 20 µg of proteins was separated on 10% SDS-PAGE gels. The results were expressed in arbitrary units after normalization for *α*-TUBULIN protein levels. The primary antibodies used were as follows: anti-DNAJB8 (diluted 1:1000 in TBST, ABclonal, China) and anti-*α*-TUBULIN (diluted 1:5000 in TBST, 11224-1-AP, Proteintech, China).

### Immunofluorescence

For immunofluorescence, the tissue sections were fixed in 4% paraformaldehyde (PFA) and blocked in 10% goat serum. The slides with spermatozoa were incubated with the following primary antibodies: anti-*γ*H2AX (diluted 1:100 in TBST, 16-202A, Merck Millipore, USA), anti-SOX9 (diluted 1:100 in TBST, AB5535, Merck Millipore, USA). Nuclear DNA and acrosome were stained with with 4′,6-diamidino-2-phenylindole (DAPI, F6057, Sigma-Aldrich, USA) and FITC-conjugated peanut agglutinin (PNA, RL-1072, Vector Labs, USA), respectively. All the spermatozoa staining was visualized on a confocal microscope (Carl Zeiss, LSM700, Germany).

### Phylogenetic analyses

Multiple amino acid sequence alignments and phylogenetic trees were constructed by the MEGA program. The amino acid sequences were downloaded from the NCBI database.

### Statistical analysis

All data are reported as mean ± SD. Significance was tested using a two-tailed unpaired Student’s t test using Prism 7.0 software. A *p* value < 0.05 was considered statistically significant. NS means not significant.

## Results

### SOX30 regulates *Dnajb8* transcription

**Figure 1:**
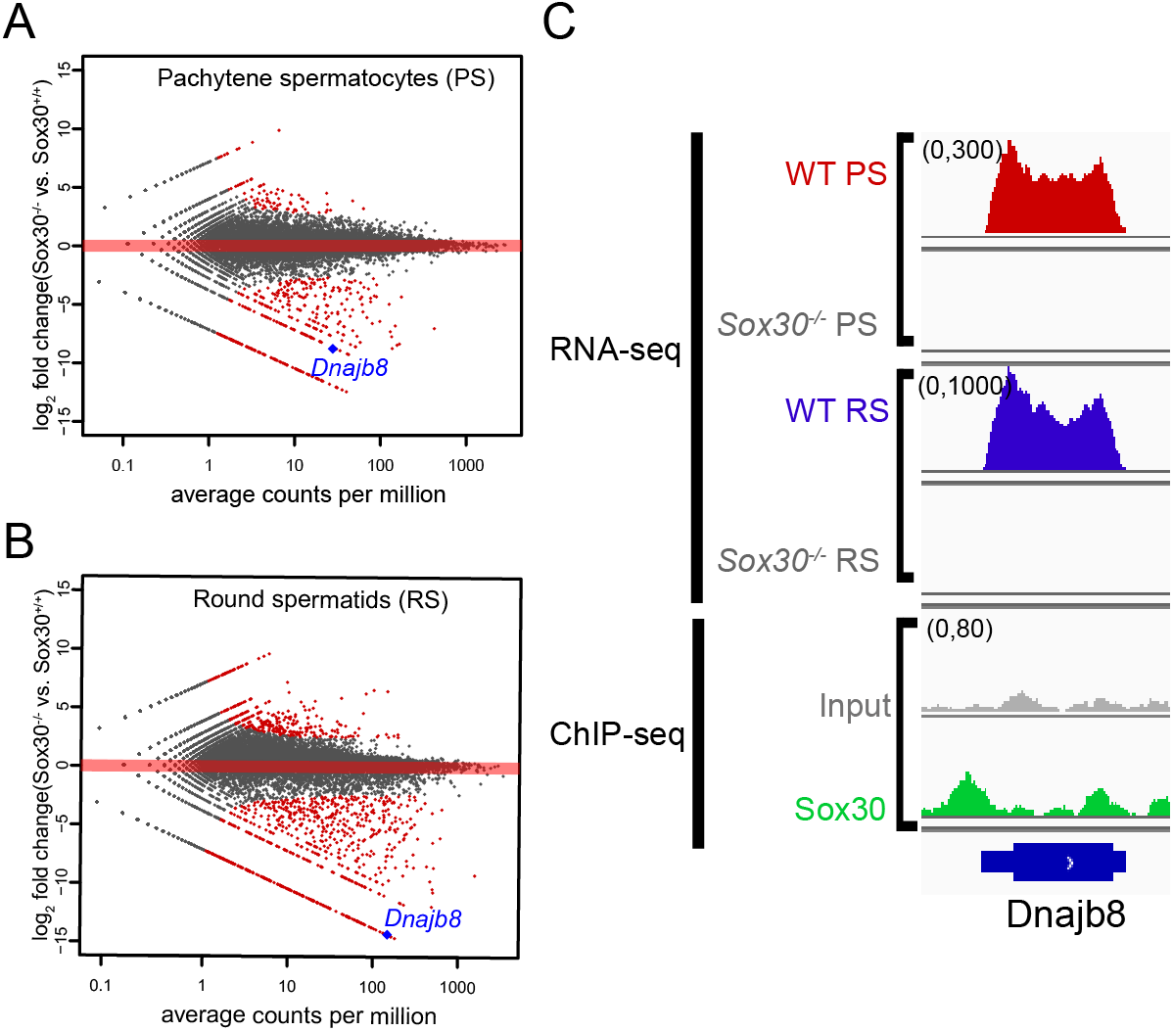
SOX30 directly regulates *Dnajb8* expression. (A, B) Scatter plot of *Dnajb8* transcripts in *Sox30* null pachytene spermatocytes and round spermatids compared with wild-type cells from published RNA-seq datasets (Bai et al., 2017). (C) Genome browser view of published SOX30 RNA-seq and ChIP-seq data on the *Dnajb8* gene loci in isolated germ cells or total testes from wild-type and *Sox30^−/−^* mice (Bai et al., 2017).

In a previous study, we combined bioinformatics analyses of the transcription factor SOX30 RNA-seq and ChIP-seq datasets to reveal that SOX30 directly regulates expression of a core set of postmeiotic genes (Bai et al., 2018). Among these direct targets, most have been reported to be involved in haploid germ cell development, such as *Tnp1*, *Hils1*, *Ccdc54* and *Tsks*, while the function of some haploid cell-enriched genes during spermatogenesis, including *Dnajb8*, remained unclear. We reanalyzed our published SOX30 RNA-seq datasets and found that *Dnajb8* was downregulated in both *Sox30* null pachytene spermatocytes and round spermatids compared with wild-type cells (Figs. 1A, 1B). In addition, our published SOX30 ChIP-seq datasets also showed that strong binding peaks were observed at the *Dnajb8* promoter, indicating that *Dnajb8* is a direct downstream target of SOX30 (Fig. 1C).

### *Dnajb8* is a conserved and testis-enriched gene

**Figure 2:**
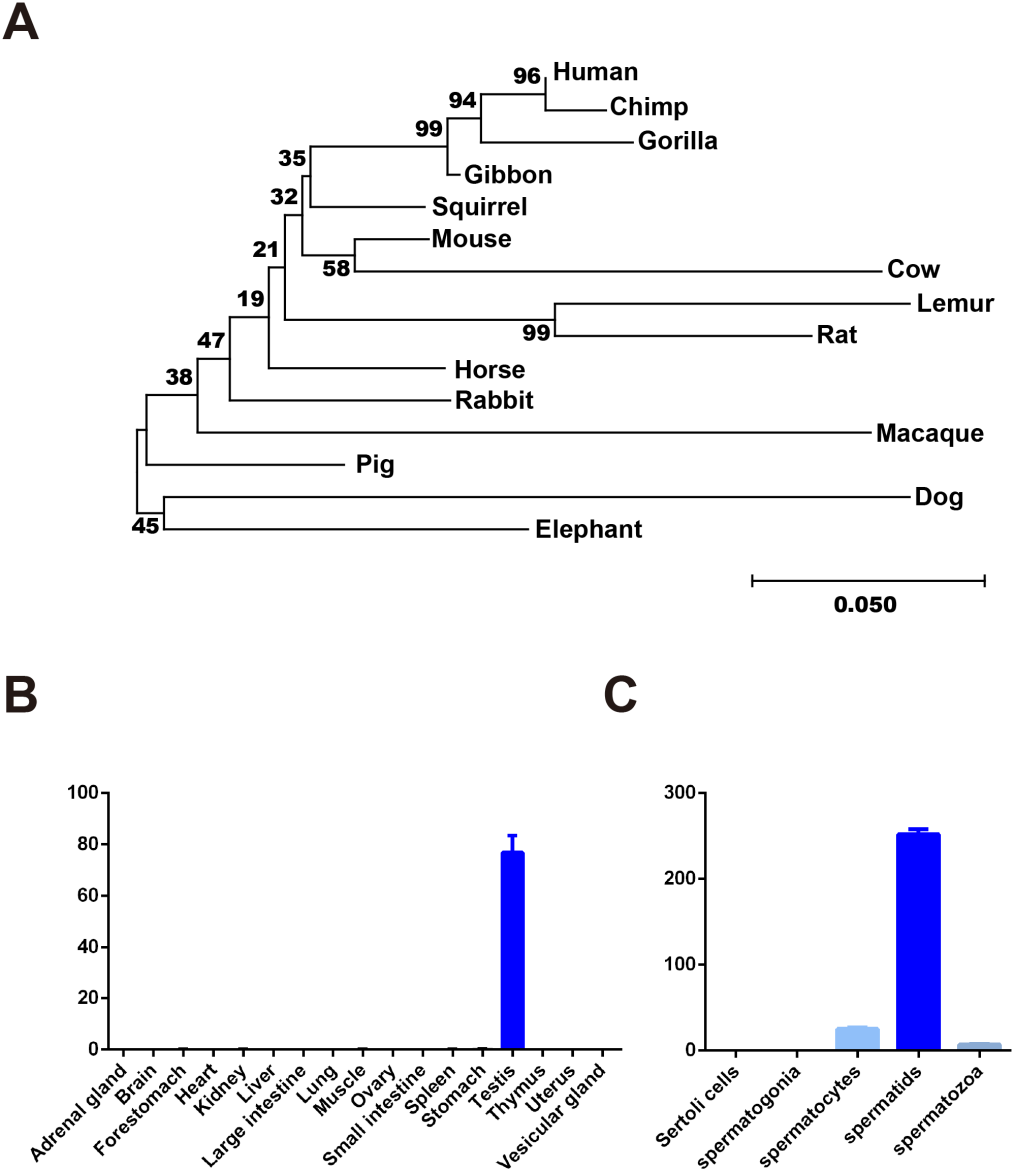
Conserved *Dnajb8* is highly expressed in mouse testis. (A) Phylogenetic trees of DNAJB8 in mammalian species. The numbers in the dendrogram were bootstrap value (%). (B, C) The median-normalized levels of *Dnajb8* mRNA expression in different mouse tissues and isolated spermatogenetic cells from published RNA-seq data (Li et al., 2017; Soumillon et al., 2013).

Phylogenetic analyses demonstrated that DNAJB8 protein is conserved between a variety of mammalian species (Fig. 2A). We further found that *Dnajb8* transcripts were highly and exclusively in the testis using published RNA-seq data generated from different mouse tissues (Fig. 2B) (Li et al., 2017). Moreover, *Dnajb8* transcripts displayed dynamic expression patterns during spermatogenesis (Soumillon et al., 2013). As shown in Fig. 2C, *Dnajb8* transcripts began to increase in pachytene spermatocytes and plateaued in round spermatids, but they were reduced in spermatozoa.

### Generation of *DNAJB8* knockout mice

**Figure 3:**
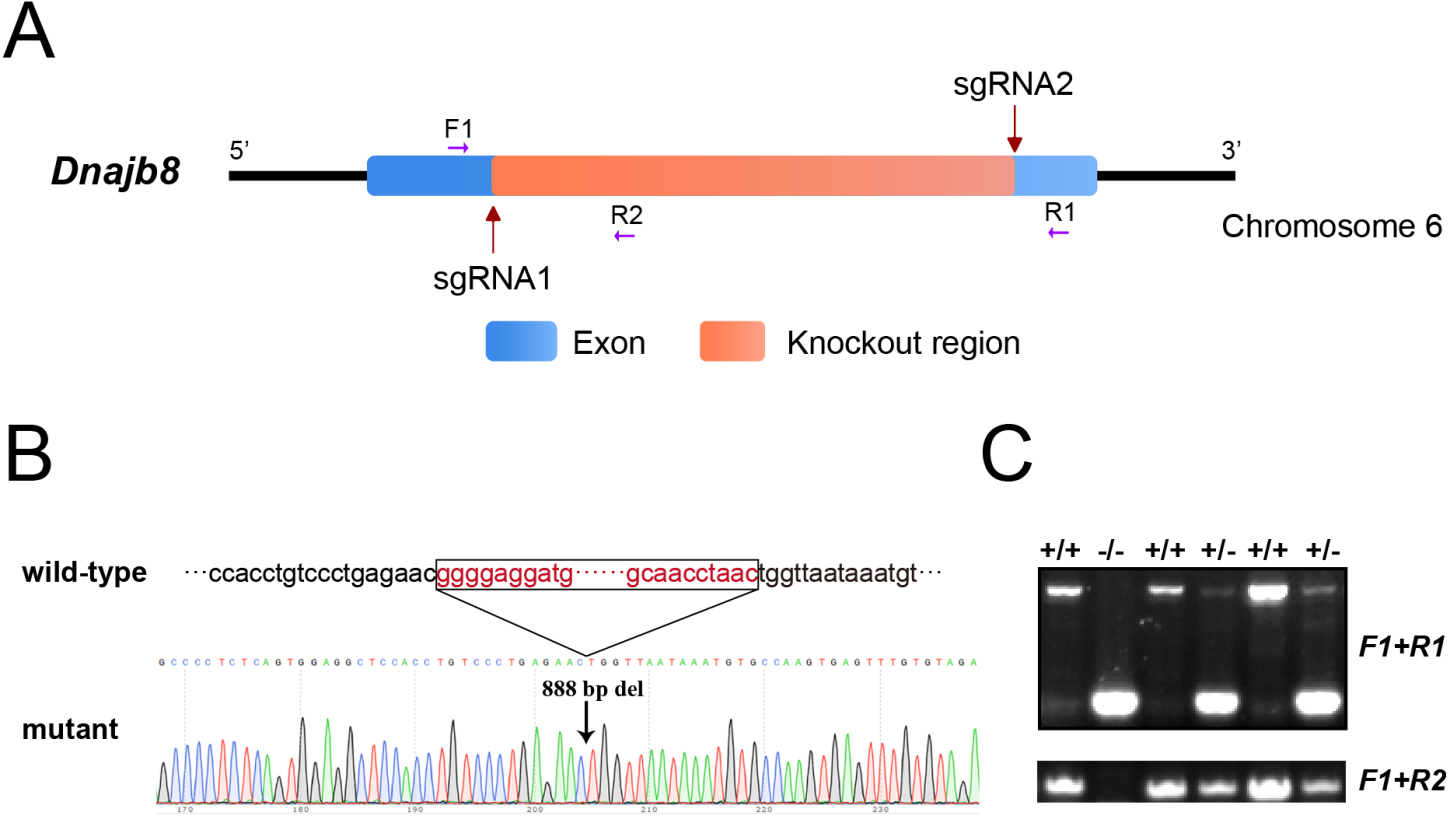
Generation of *Dnajb8*-null mice by the CRISPR/Cas9 system. (A) Schematic illustration of the generation of *Dnajb8*^−∕−^ mice. Two sgRNAs were designed to target the 5′ and the 3′ region respectively of the coding exon. Three primers (F1, R1 and R2) were designed for genotyping. (B) Sanger sequencing from wild-type and *Dnajb8*^−∕−^ mice. A 888-bp deletion were detected in *Dnajb8*^−∕−^ mice. (C) Genotype verification of *Dnajb8*^−∕−^ mice by genomic PCR using primer sets F1-R1 and F1-R2, respectively.

To explore the function of DNAJB8, *Dnajb8* mutant mice with a *Danjb8* allele containing an 888-bp deletion were generated by CRISPR-Cas9 system (Figs. 3A, 3B). Homozygous mutant *Dnajb8* alleles were obtained by selective breeding and determined by genotyping PCR (Fig. 3C).

### *Dnajb8* deficient male mice are fertile

**Figure 4:**
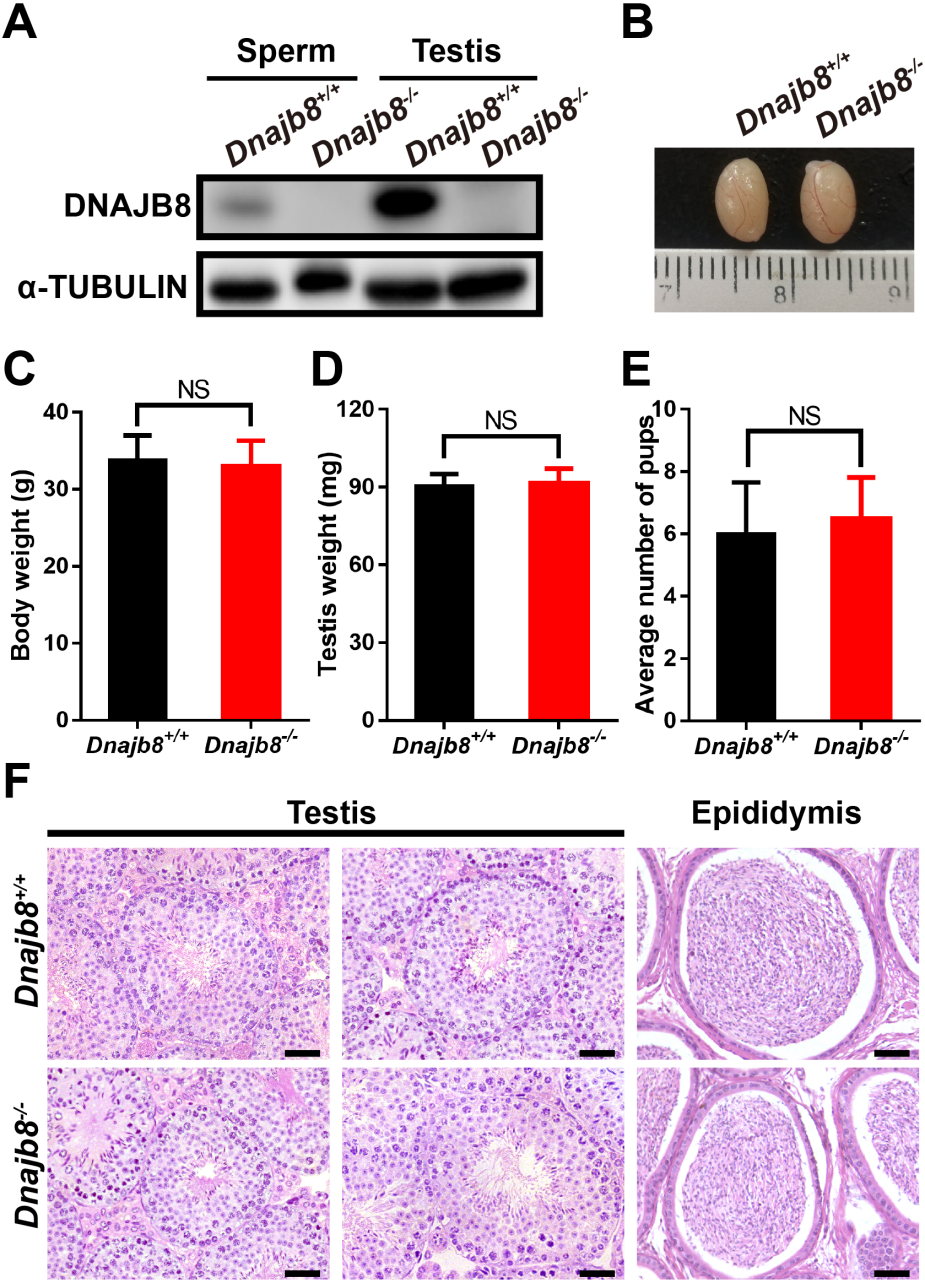
*Dnajb8*^−/−^ mice are fertile. (A) Western blot conformed that DNAJB8 protein was absent in adult *Dnajb8*^−∕ −^ testes and sperm. (B) The morphology of wild-type and *Dnajb8*-null testes at 10-week-old. (C, D) The body weight (C) and testis weight (D) from *Dnajb8*^+∕+^ and *Dnajb8*^−/−^ mice at 10-week-old. *Dnajb8*^+∕+^, *n* = 6; *Dnajb8*^−/−^, *n* = 5. (E) Number of pups per litter from *Dnajb8*^+∕+^ and *Dnajb8*^−/ −^ males. Each genotype shown was coupled with *Dnajb8*^+∕+^ females. *n* = 3 for each genotype. (F) H&E staining of testes and epididymis from adult *Dnajb8*^+∕+^ and *Dnajb8*^−/−^ mice at 10-week-old. Scale bar: 50 µm. NS, No significant difference.

Western blot analysis confirmed the absence of DNAJB8 protein in testis and sperm from adult *Dnajb8*^−∕−^ mice (Fig. 4A). All *Dnajb8*^−∕−^ males were viable and phenotypically normal. The body and testicular weight of adult *Dnajb8*^−∕−^ males (*n* = 5) was comparable to that of wild-type males (*n* = 6) at 10-week-old (Figs. 4B–4D). Mating tests showed normal litter sizes from *Dnajb8*^−∕−^ males (Fig. 4E). Moreover, complete spermatogenesis in seminiferous tubules and normal epididymal structures were observed in *Dnajb8*^−∕−^ mice by H&E staining (Fig. 4F).

### Normal sperm parameters in *Dnajb8*^−∕−^ mice

**Figure 5:**
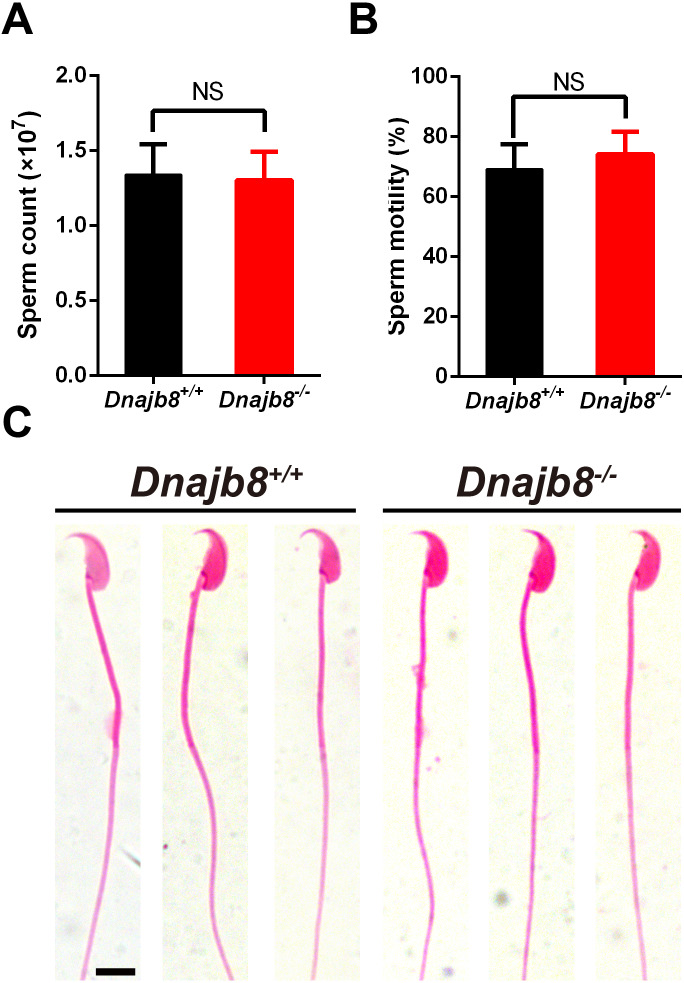
Normal sperm count, morphology and motility in *Dnajb8*^−/ −^ mice. (A, B) Sperm count and motility of the cauda epididymal from *Dnajb8*^+∕+^ and *Dnajb8*^−/ −^ mice at 10-week-old. *Dnajb8*^+∕+^, *n* = 6; *Dnajb8*^−/ −^, *n* = 5. (C) Sperm morphology from *Dnajb8* ^+∕+^ and *Dnajb8*^−/ −^ mice by H&E staining at 10-week-old. *n* = 3 for each genotype. Scale bar: 10 µm. NS, No significant difference.

The number and motility of mature spermatozoa from the epididymal cauda of *Dnajb8*^−∕−^ mice (*n* = 5) were similar with those of *Dnajb8*^+∕+^ controls (*n* = 6) at 10-week-old (Figs. 5A, 5B). In addition, sperm from *Dnajb8*^−∕−^ males exhibited normal morphology (Fig. 5C).

### DNAJB8 is not essential for germ cell development

**Figure 6:**
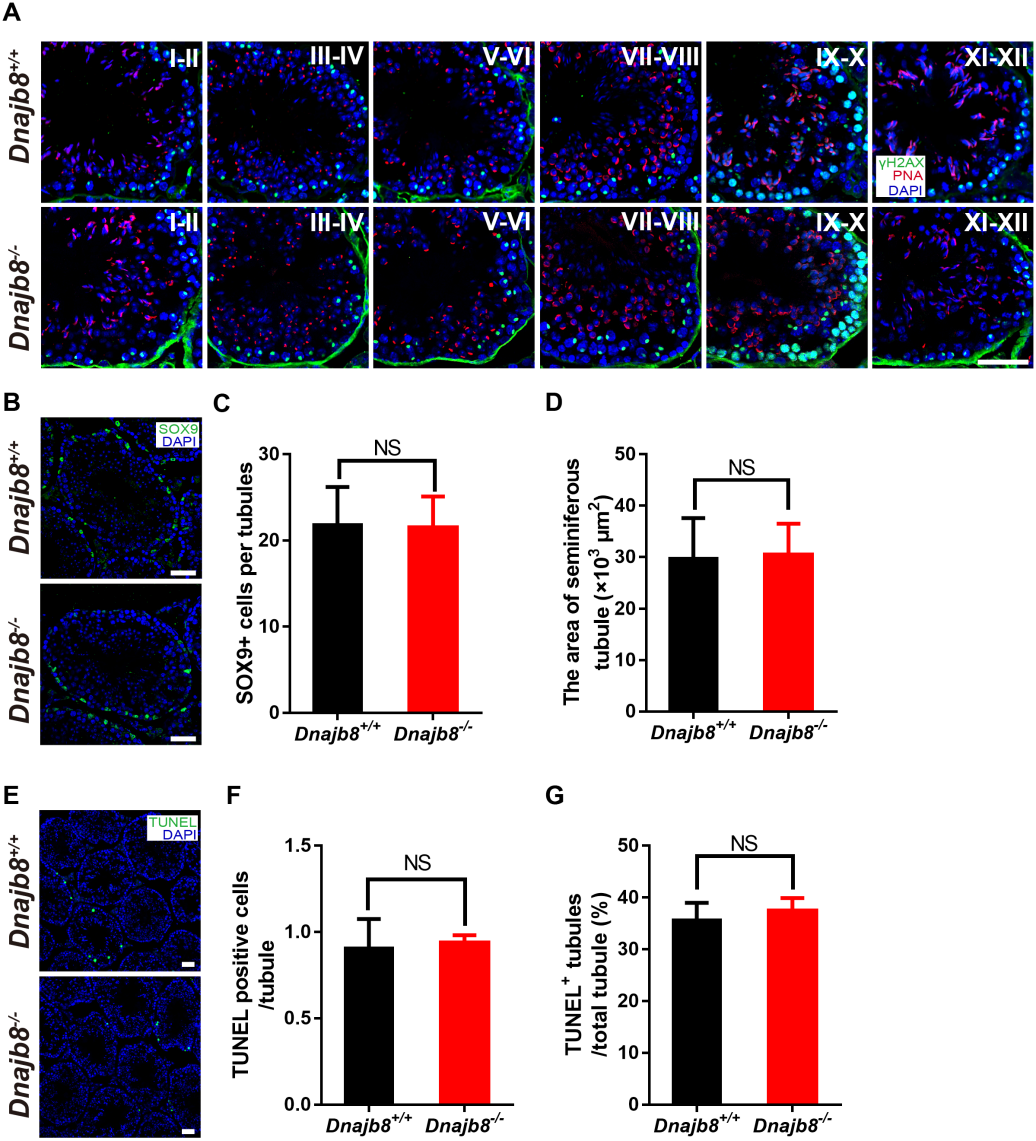
*Dnajb8^-/-^* males show normal spermatogenesis. (A) Immunodetection of *γ*H2AX and PNA in testis sections from adult *Dnajb8*^+∕+^ and *Dnajb8*^−/ −^ mice. Scale bar: 50 µm. (B) Immunostaining of adult testis sections for SOX9, a marker of Sertoli cells. Scale bar: 50 µm. (C) Quantification of SOX9-positive cells in seminiferous tubule from *Dnajb8*^+∕+^ and *Dnajb8*^−/ −^ mice at 10-week-old. Total 60 tubules from three independent mice per group were counted. (D) Seminiferous tubule area in *Dnajb8*^+∕+^ and *Dnajb8*^−/ −^ testes at 10-week-old. Total 60 tubules from three independent mice per genotype were analyzed. (E) TUNEL assay on testis sections from adult *Dnajb8*^+∕+^ and *Dnajb8*^−/ −^ mice at 10-week-old. Scale bar: 50 µm. (F) Quantification of TUNEL-positive cells per tubule. (G) The ratios of TUNEL-positive tubules to total tubules in *Dnajb8*^+∕+^ and *Dnajb8*^−/−^ testes. Total 157 and 168 from three independent mice were counted per genotype, respectively. NS, No significant difference.

To characterize the spermatogenesis in *Dnajb8*^−∕−^ mice, we next performed immunostaining for *γ*-H2AX-positive spermatocytes and PNA-positive acrosomes in spermatids. As shown in Fig. 6A, different stages of spermatocytes and spermatids were both observed in seminiferous tubules from adult wild-type and *Dnajb8*^−∕−^ mice. We then immunostained testis sections for the Sertoli cell marker SOX9 and found that seminiferous tubules from wild-type and *Dnajb8*^−∕−^ males (*n* = 3 for each group) contained similar numbers of Sertoli cells (Figs. 6B, 6C). The area of seminiferous tubules from *Dnajb8*^+∕+^ and *Dnajb8*^+∕+^testes (*n* = 3 for each group) were similar (Fig. 6D). Furthermore, TUNEL staining revealed that the numbers of apoptotic cells and tubules did not differ between *Dnajb8*^−∕−^ and *Dnajb8*^+∕+^ testes (*n* = 3 for each group) (Figs. 6E–6G). These results show that *Dnajb8* deficiency does not affect spermatogenesis in *Dnajb8*^−∕−^mice.

## Discussion

Here, we reanalyzed RNA-seq and ChIP-seq datasets from the transcription factor SOX30 and demonstrated that SOX30 directly regulates expression of the postmeiotic gene *Dnajb8*. As *Dnajb8* is a highly conserved and testis-enriched gene, we further generated *Dnajb8* KO mice by CRISPR/Cas9 and found that *Dnajb8* is not required for male fertility, with no difference in testicular or epididymal histology and average litters sizes compared to wild-type males.

To date, several DNAJB proteins have been identified as being involved in spermatogenesis and male fertility (Meccariello et al., 2014). DNAJB1 is mainly expressed in mouse testis and is localized in the acrosomal region of the sperm head, the middle and end pieces of the sperm tail (Doiguchi et al., 2007). DNAJB3 protein is highly expressed in haploid germ cells and may be involved in vesicle fusion (Berruti & Martegani, 2001). Importantly, it has been reported that homozygous mutations in DNAJB13, a radial spoke protein of the mouse ‘9+2’ axoneme that localized to the sperm flagella, cause male infertility, with severe oligo-astheno-teratozoospermia (El Khouri et al., 2016; Li & Liu, 2014). Moreover, heterozygous variants in DNAJB13 were correlated with male fertility in asthenozoospermia (Li et al., 2020). DNAJB13 interactions with SUN5 play a crucial role in sperm head-tail integration (Shang et al., 2018). Similar to the expression pattern of the DNAJB family, *Dnajb8* is a testis-specific gene and is predominantly in spermatids. Notably, *Dnajb8* was downregulated in spermatozoa of infertile men by 12-fold compared to that of normospermic individuals (Montjean et al., 2012). Hence, DNAJB8 was thought to play a role in germ cell development. However, our study showed that all stages of spermatogenic cells were detected in *Dnajb8*^−∕−^ seminiferous tubules. Meanwhile, immunofluorescence staining of *γ*H2AX-positive spermatocytes and PNA-positive spermatids, as well as SOX9-positive Sertoli cells, were not different between *Dnajb8*^−∕−^ and *Dnajb8*^+∕+^ mice. These data indicate that DNAJB8 protein is not essential for spermatogenesis.

Previous studies have found that more than 54 conserved testis-enriched proteins were not essential for fertility (Miyata et al., 2016). This may be because these genes are highly covered by the redundancy of other genes. Functional redundancy of DNAJ family proteins has been shown to exist in yeast (Sahi & Craig, 2007). Interestingly, most *Dnajb* transcripts were mainly detected in the testis, especially the haploid germ cell-enriched genes *Dnajb3* and *Dnajb7* (Li et al., 2017; Soumillon et al., 2013), indicating that redundancy may be found in human DNAJs. Thus, DNAJ proteins work in parallel, and this redundancy could explain the normal male fertility of *Dnajb8* KO mice.

Although we investigated litter size using co-housing assay, which provides unrestricted access of males to females, the protocol may be not detect subtle changes in *Dnajb8* KO males. Nevertheless, we have shown that *Dnajb8* is not essential for male fertility under normal laboratory mating conditions in this study.

Taken together, our findings demonstrate that mouse DNAJB8 is dispensable for spermatogenesis and male fertility. Even though *Dnajb8* is a postmeiotic gene directly regulated by SOX30, we did not observe any defects in germ cell development in *Dnajb8*^−∕−^ males. One possible explanation for this is the redundant mechanisms of DNAJ proteins that control male fertility.

##  Supplemental Information

10.7717/peerj.10582/supp-1 Supplemental Information 1 Raw numerical data

10.7717/peerj.10582/supp-2 Supplemental Information 2 Raw data for Fig. 3C

10.7717/peerj.10582/supp-3 Supplemental Information 3 Raw data for Fig. 4A

## Additional Information and Declarations

### DNA Deposition

The following information was supplied regarding the deposition of DNA sequences: SOX30 RNA-seq and ChIP-seq data are available at NCBI SRA: PRJNA433934 (accession: SRP143508).

### Data Availability

The following information was supplied regarding data availability: The raw measurements are available in the Supplemental Files.
