# Supplementary figures and images for: Dnajb8, a target gene of SOX30, is dispensable for male fertility in mice
Source: PeerJ. 2020 Dec 21;8:e10582. doi: 10.7717/peerj.10582 (PMC7759119; doi:10.7717/peerj.10582)

M

- 1318 bp

- 430 bp

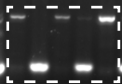

M

520 bp -

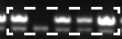

Supplement: Supplemental Information 2 [file peerj-08-10582-s002.pdf]
