# Supplementary material for: Dnajb8, a target gene of SOX30, is dispensable for male fertility in mice
Source: PeerJ. 2020 Dec 21;8:e10582. doi: 10.7717/peerj.10582 (PMC7759119; doi:10.7717/peerj.10582)
Supplement: Supplemental Information 3 [file peerj-08-10582-s003.pdf]

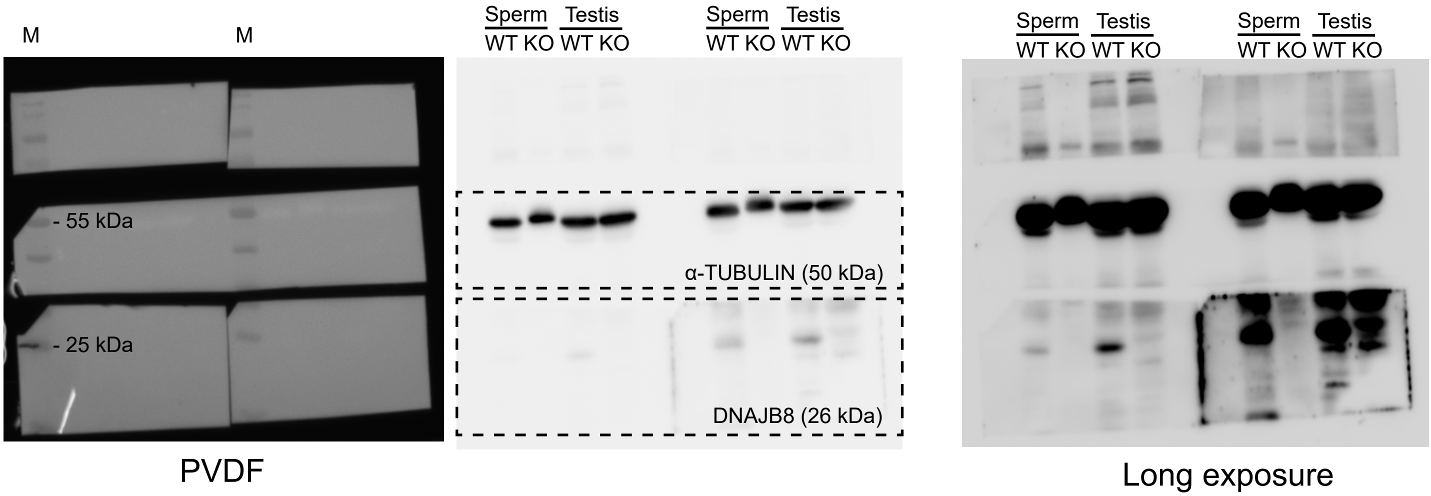
M
M
- 55 kDa
- 25 kDa
PVDF
Sperm
Testis
WT KO WT KO
Sperm
Testis
WT KO WT KO
α-TUBULIN (50 kDa)
DNAJB8 (26 kDa)
Sperm
Testis
WT KO WT KO
Sperm
Testis
WT KO WT KO
Long exposure
